# Perivascular Adipose Tissue-Derived Adiponectin Inhibits Collar-Induced Carotid Atherosclerosis by Promoting Macrophage Autophagy

**DOI:** 10.1371/journal.pone.0124031

**Published:** 2015-05-28

**Authors:** Changlong Li, Zhijian Wang, Chunxiao Wang, Qian Ma, Yingxin Zhao

**Affiliations:** 1 Beijing Institute of Heart Lung and Blood Vessel Diseases, Beijing, China; 2 Department of Cardiology, Anzhen Hospital, Capital Medical University, Beijing, China; Tohoku University, JAPAN

## Abstract

**Objectives:**

Adiponectin (APN) secreted from perivascular adipose tissue (PVAT) is one of the important anti-inflammatory adipokines to inhibit the development of atherosclerosis, but the underlying mechanism has not been clarified. In this study, we aimed to elucidate how APN regulates plaque formation in atherosclerosis.

**Methods and Results:**

To assess the role of APN secreted by PVAT in atherosclerosis progression, we performed PVAT transplantation experiments on carotid artery atherosclerosis model: ApoE knockout (*ApoE−/−*) mice with a perivascular collar placement around the left carotid artery in combination with a high-fat diet feeding. Our results show that the *ApoE^−/−^* mice with PVAT derived from APN knockout (APN^−/−^) mice exhibited accelerated plaque volume formation compared to *ApoE^−/−^* mice transplanted with wild-type littermate tissue. Conversely, autophagy in macrophages was significantly attenuated in *ApoE^−/−^* mice transplanted with APN^-/-^ mouse-derived PVAT compared to controls. Furthermore, *in vitro* studies indicate that APN treatment increased autophagy in primary macrophages, as evidenced by increased LC3-I processing and Beclin1 expression, which was accompanied by down-regulation of p62. Moreover, our results demonstrate that APN promotes macrophage autophagy via suppressing the Akt/FOXO3a signaling pathway.

**Conclusions:**

Our results indicate that PVAT-secreted APN suppresses plaque formation by inducing macrophage autophagy.

## Introduction

Atherosclerosis is a complex chronic inflammatory and metabolic disease, which is a major contributor of morbidity and mortality in the world. In addition to lipid dysfunction and arterial lipid accumulation, immune-inflammatory response has been increasingly recognized as essential reason in atherogenesis [1, 2]. Macrophages are largely accumulated in atherosclerotic plaques and play crucial roles in atherosclerotic immune responses [3]. Emerging evidence suggests that macrophage autophagy exerts protective role in atherosclerosis [4, 5], which has demonstrated a novel pathway to therapeutically suppress atherosclerosis progression [6, 7].

Several autophagy triggers are present in the atherosclerotic plaque, such as inflammatory mediators, ROS production and accumulation of oxidized LDL [8, 9]. Recent study has reported that adiponectin (APN) could modulate the activation of autophagy in vitro and in vivo [10, 11]. Adiponectin is one of several important, metabolically active cytokines secreted from adipose tissue, which exerts bio-effects on multiple type of cells and has anti-inflammatory and anti-atherosclerotic properties [12]. Previous studies have demonstrated that APN inhibits atherosclerosis by suppressing atherogenic processes within the blood vessel wall [13, 14]. However, the precise mechanism by which APN regulates anti-atherosclerotic responses and macrophages function in atherosclerosis remains to be revealed.

In addition to visceral adipose tissue, perivascular adipose tissue (PVAT) secretes a great deal of APN that can act in both autocrine and paracrine fashion [15]. Although PVAT can support inflammation during atherosclerosis through macrophage accumulation, recent reports reveal that PVAT also has anti-atherosclerotic properties related to its abilities to secrete anti-inflammatory adipokines [16, 17]. These paradoxical findings suggest that differences in either the type or level of a particular PVAT-derived adipokine may determine its role in atherosclerosis development. However, the molecular mechanisms maintaining that balance have not been fully identified.

In the present study, we investigated the role of PVAT-derived APN in collar-induced carotid atherosclerosis and the molecular mechanism involved in the regulation of macrophage autophagy. Our results indicate that PVAT-derived APN deficiency increased plaque volume formation in *ApoE* ^*−/−*^ mice when compared with wild-type control with sufficient PVAT-derived APN. This was associated with decreased autophagy in vascular macrophages. These results suggest that PVAT derived-APN contributes to inhibition of plaque formation by inducing macrophage autophagy.

## Materials and Reagents

### Antibodies and reagents

Anti-phosphor-FOXO3a, anti-FOXO3a, anti-PTEN and anti-β-actin were purchased from Santa Cruz Biotechnology (Santa Cruz, CA). Anti-LC3 was obtained from Abcam. Anti-phosphor-AKT, anti-AKT, Anti-phosphor-mTOR and anti-mTOR were purchased from Cell Signaling Technology (Beverly, MA). Recombinant globular APN was purchased from BioVision (Mountain View, CA).

### Animal model and adipose tissue transplantation

Male APN^-/-^ mice were purchased from the Jackson Laboratory. Male *ApoE* ^*-/-*^ mice and wild type mice were purchased from Peking University (Beijing, China). All mice were 8 weeks old and in C57BL/6J background.

Mice underwent perivascular collar placement after deep anesthesia with an intraperitoneal injection of pentobarbital sodium. As described previously [18], a constrictive perivascular silica collar (0.3 mm in internal diameter and 3 mm in length) was placed around the left carotid artery. Animals were fed for 12 weeks and kept on a 12 h light/12 h dark cycle. All mice received a high-fat diet (D12492 from Vital River Laboratory) throughout the experiment.

In addition, to analyze the effects of APN secreted by PVAT on atherosclerotic plaque disruption, we administered lipopolysaccharide (LPS) into ApoE-/- mice after collar replacement [19]. Four weeks after surgery, mice in the LPS groups were injected intraperitoneally with LPS (1 mg/kg, Sigma) twice a week for 8 weeks.

The adipose tissue transplantation was performed as described previously [20]. Atherosclerosis model was performed on left carotid artery with or without perivascular adipose tissue transplantation. 10 mg of perivascular adipose tissue was harvested respectively from APN^-/-^ mice and corresponding wild-type counterparts. The adipose tissue was implanted around the site of carotid artery using 9–0 Nylon after removal of endogenous PVAT. The mice transplanted with wild-type and APN^-/-^ adipose tissue were respectively named as (WT) PVAT and (KO) PVAT. All procedures were approved by the Animal Care and Use Committee of Capital Medical University.

### Hematoxylin and eosin (H&E) staining

Mouse hearts were perfused with saline. The carotid artery was isolated and fixed with 4% paraformaldehyde for 30 min, embedded in paraffin and cut into 5 μm serial sections. In brief, corresponding sections were stained with hematoxylin for 4 min. Subsequently, the sections were washed with 1% hydrochloric acid alcohol differentiation liquid for 5 s and washed with running water for 5 min. Sections were then stained with eosin for 4 min. Images were captured by Nikon Eclipse TE2000-S microscope (Nikon, Tokyo, Japan) and analyzed by Image Pro Plus 3.1 (Nikon).

### Immunofluorescence

The carotid artery were embedded in OCT embedding medium and cut into 7μm serial sections as described. Sections were blocked using 5% fetal bovine serum for an hour. Then the sections were stained with primary antibodies (1:500) or IgG instead of primary antibody as negative control overnight at 4°C. After incubation with FITC- or tetra-methylrhodamineisothiocyanate-conjugated secondary antibodies (Jackson ImmunoResearch Laboratories) (1:100) at room temperature for 1hour. Sections were observed by Nikon Eclipse TE2000-S microscope (Nikon, Tokyo, Japan) and analyzed by Image Pro Plus 3.1 (Nikon).

### Primary smooth muscle cell and macrophage culture

Isolation of the primary smooth muscle cells from the arteries was performed as described previously [21]. SMCs were grown in F12/DMEM supplemented with 20% fetal bovine serum (FBS), monothioglycerol (1.2 mmol/L), L-glutamine (2 mmol/L), penicillin (100 U/mL) and streptomycin (100 μg/mL). Bone marrow-derived macrophages were prepared as described previously with minor modification [22]. Myeloid origin macrophage were plated in DMEM complete medium (10% FCS, 50 U/mL penicillin, 50 μg/mL streptomycin) supplemented with murine macrophage colony-stimulating factor (M-CSF, 50 ng/mL) cultured for 5 days. All the Cells were grown with 5% CO_2_ at 37°C.

### Western blot

Proteins were extracted from three carotid artery. Western blot analysis was performed as described [23]. In briefly, 50 μg protein lysates were separated by 15% SDS-PAGE and transfered to nitrocellulose membranes (Millipore). The blots were incubated with the primary antibodies (1:1000) at 4°C overnight, and then with infrared Dye-conjugated secondary antibodies (1:10000; Rockland Immunochemicals) for 1 hr at 37°C. The images were quantified by the use of the Odyssey infrared imaging system (LI-COR Biosciences, Lincoln, NE, USA).

### Statistical analysis

All data are presented as mean ± SEM. Differences between groups were analyzed using the Student's t test by Newman-Keuls multiple comparison test from GraphPad Prism (GraphPad Software). P<0.05 was considered statistically significant.

## Results

### APN deficiency in perivascular adipose tissue aggravated atherosclerosis development in *ApoE* ^*-/-*^ mice

**Fig 1:**
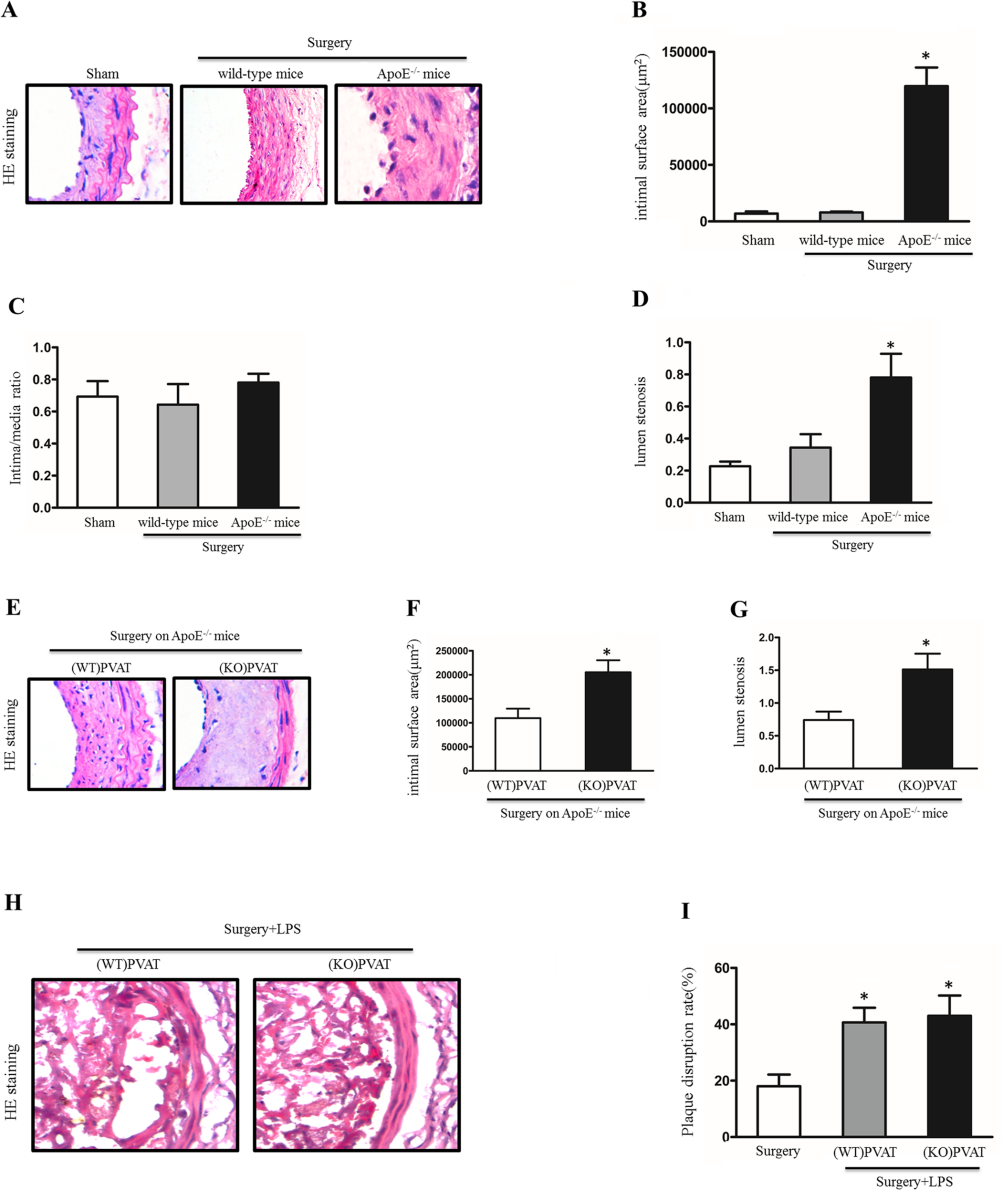
APN derived from PVAT protects mice from atherosclerosis. A, HE staining of arteria carotis sections from mice after perivascular collar placement for 12 weeks. B, Quantitative analysis of intimal surface area in arteria carotis of each groups (n = 6 per group). *P<0.05 versus sham. C, Analysis of intima/media ratio in arteriacarotis with each groups (n = 6 per group). D, Bar graph shows quantification of lumen stenosis in arteria carotis with each groups (n = 6 per group). *P<0.05 versus sham. E, HE staining of arteria carotis sections from ApoE-/- mice transplanted with WT or APN-/- PVAT 12 weeks after atherosclerosis established. F, Quantitative analysis of intimal surface area in arteria carotis with WT or APN-/- PVAT (n = 6 per group). *P<0.05 versus (WT) PVAT. G, Bar graph shows quantification of lumen stenosis in arteria carotis with WT or APN-/- PVAT (n = 6 per group). *P<0.05 versus (WT) PVAT. H, HE staining of atherosclerotic plaques from the indicated mice. I, plaque disruption rate of he indicated mice (n = 6 per group). *P<0.05 versus surgery control.

To examine the effects of APN secreted by perivascular adipose tissue on the development of atherosclerosis, we firstly performed the site-controlled atherogenesis on the C57/BL6 wild-type mice and the *ApoE* ^*-/-*^ mice by perivascular collar placement. 12th weeks after collar insertion, a significant increase in intimal surface area had occurred in the *ApoE* ^*-/-*^ mice, but the intimal surface area did not rise significantly in the corresponding sites of the C57/BL6 wild-type mice (Fig 1A and 1B). The degree of lumen stenosis was higher in collar insertion sites of the *ApoE* ^*-/-*^ mice compared with surgical animals of the C57/BL6 wild-type mice (Fig 1C). But, the collar insertion sites did not display a significant increase in intima-media ratio in either groups (Fig 1D).

Next, we performed the perivascular adipose tissue transplantation experiments on the *ApoE* ^*-/-*^ mice after perivascular collar placement. We removed PVAT around the left carotid artery after perivascular collar placement and transplanted PVAT from APN^-/-^ mice or wild-type counterparts. HE staining revealed that mice transplanted with PVAT from APN^-/-^ mice had an aggravated plaque and lumen stenosis compared with mice transplanted with wild-type tissue (Fig 1E and 1G). Intimal surface area measurement showed that the intimal surface area in mice with APN^-/-^ tissue obviously increased in comparison with mice with wild-type tissue (Fig 1F).

Finally, to analyze the effects of APN secreted by PVAT on atherosclerotic plaque disruption, we administered lipopolysaccharide (LPS) into *ApoE* ^*-/-*^ mice after collar replacement. As shown in Fig 1H and 1I, the disruption rates in LPS groups were significantly higher than those of controls, but there is no difference in the disruption rates between the *ApoE* ^*-/-*^ mice transplanted with APN^-/-^ tissue and wild-type tissue. Taken together, these results indicated that APN derived from PVAT restricted atherosclerosis development in *ApoE* ^*-/-*^ mice, but didn’t affect atherosclerotic plaque disruption.

### Deficiency of PVAT-derived APN reduced autophagy in plaque

**Fig 2:**
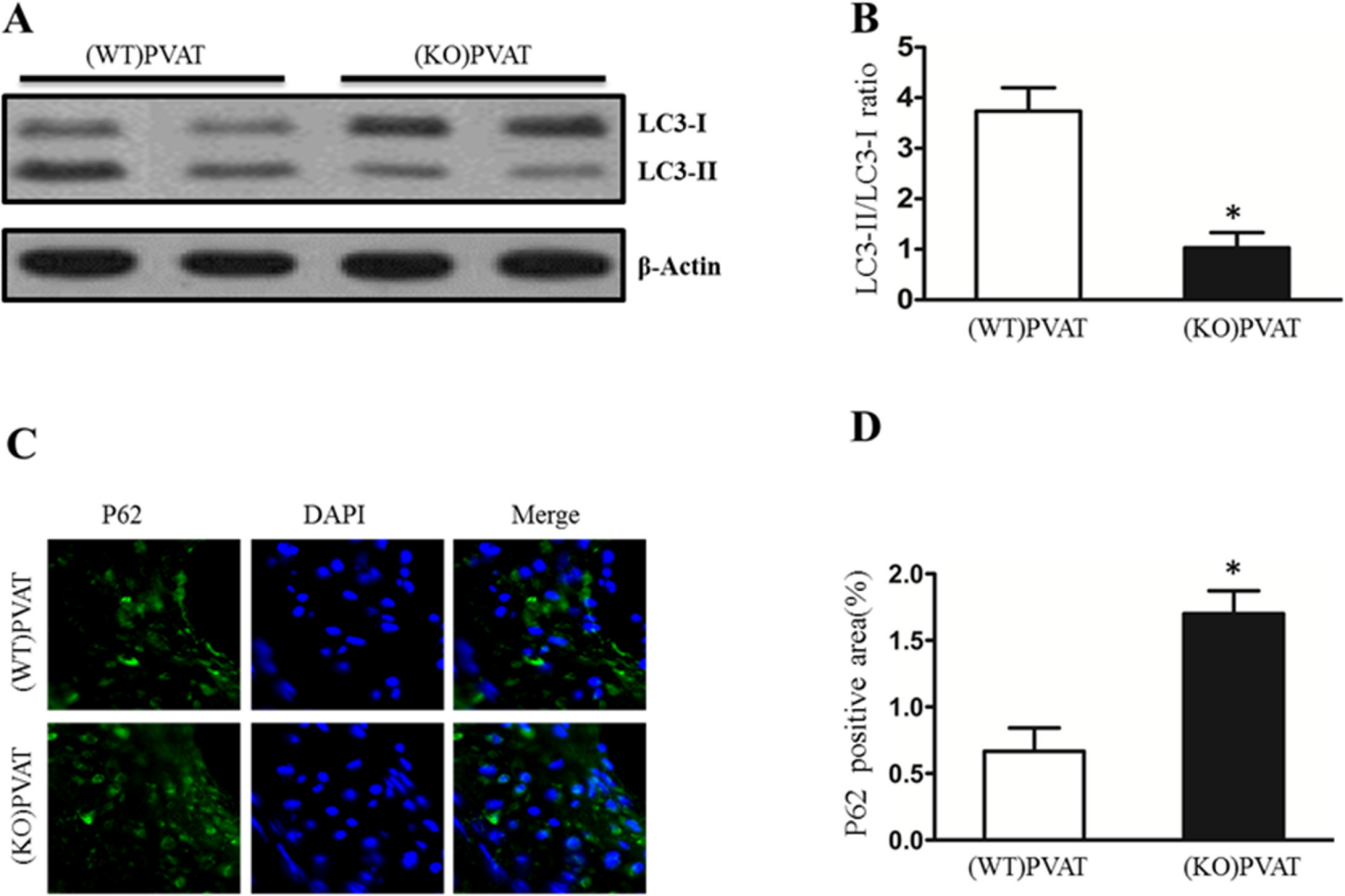
APN secreted by PVAT aggravates autophagy in plaque. A, Representative western blot of LC3 expression in arteria carotis transplanted with WT or APN^-/-^ PVAT 4 weeks after atherosclerosis (n = 6 per group). B, Quantitative analysis of LC3 protein expression in various groups. *P<0.05 versus (WT) PVAT. C, Immunofluorescence of p62, another marker of autophagy, in the arteria carotis with WT or APN^-/-^ PVAT (n = 6 per group). D, Histogram shows p62 positive cells per 100 cells. *P<0.05 versus (WT) PVAT.

To investigate the role of autophagy in atherosclerosis, we compared the LC3-II level in the arteriacarotis. Western blot revealed that the protein level of LC3-II in arteriacarotis of mice transplanted with APN^-/-^ tissue is lower than mice transplanted with wild-type tissue (Fig 2A and 2B). To directly observe the difference of autophagy in both group, we performed an immunofluorescence staining of p62 on arteriacarotis. Immunofluorescence assay showed that APN deficiency increased the accumulation of p62 in plaque of *ApoE* ^*-/-*^ mice (Fig 2C and 2D). These results demonstrate that PVAT-derived APN promotes autophagy in plaque.

### APN aggravated autophagy on macrophages

**Fig 3:**
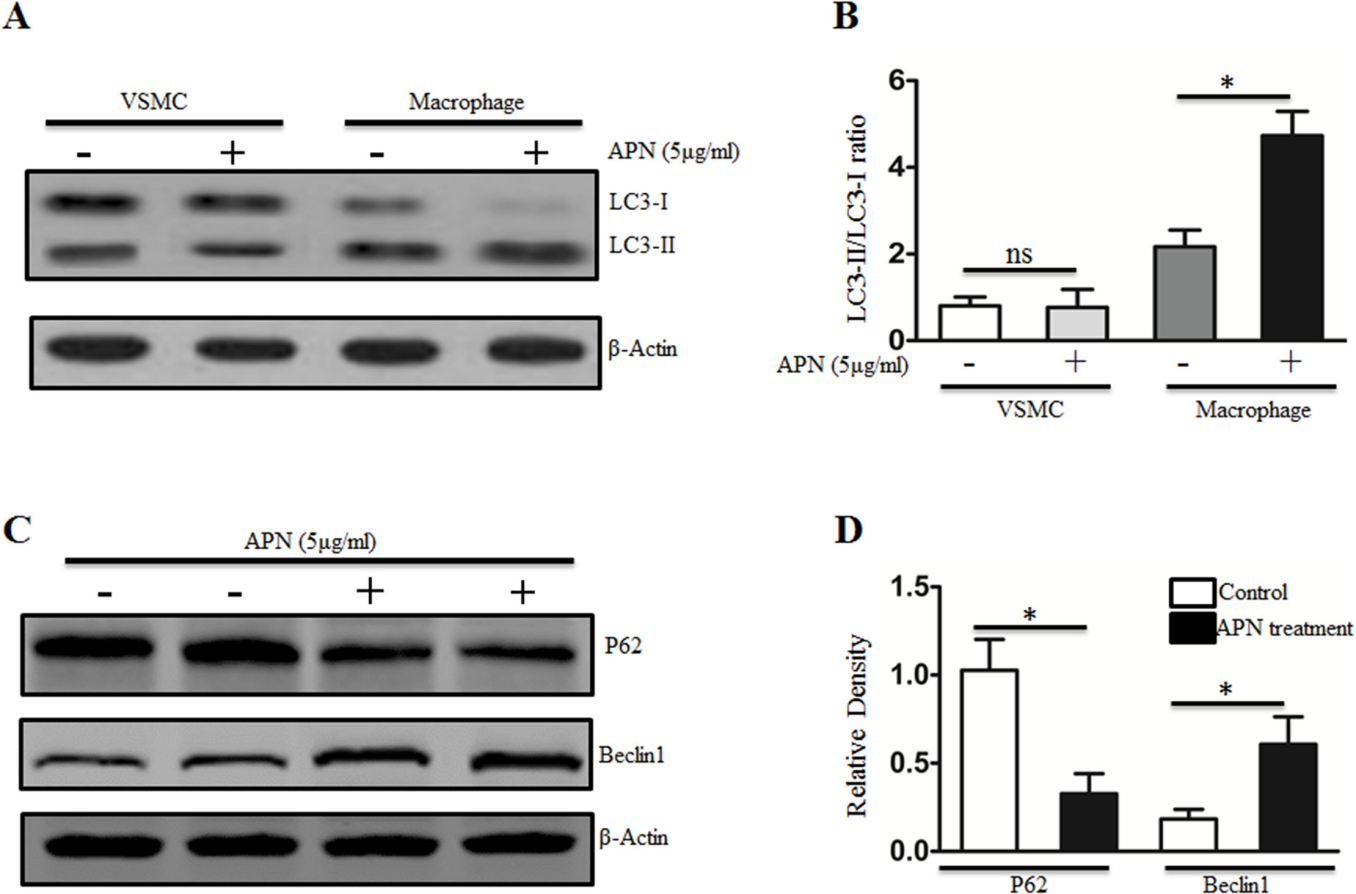
APN exacerbates macrophage autophagy in vitro. A and B respectively show the representative western blot and quantitative analysis of LC3 protein level in VSMC and macrophage stimulated with or without APN (5 μg/ml). n = 6 per group. *P<0.05 versus macrophage without APN. C and D respectively show the western blot and quantitative analysis of P62 and Beclin 1 protein level in macrophage treated with or without APN (5 μg/ml). n = 6 per group. *P<0.05 versus macrophage without APN.

Since autophagy existed on both macrophages and smooth muscle cells in the lesions [24], we examined the autophagy response in these cells. As shown in Fig 3A, the LC3-I processing was significantly increased in macrophages stimulated by APN. To further ascertain the role of APN in macrophage, we assessed the protein level of Beclin1 and p62, the other markers of autophagy. Western blot showed that APN treatment increased the levels of Beclin1 in macrophages, which was associated with down-regulation of p62 (Fig 3B). These results demonstrate that APN obviously promotes the autophagy in macrophage, but not in smooth muscle cells.

### APN aggravated macrophage autophagy through the Akt/FOXO3a signaling pathway

**Fig 4:**
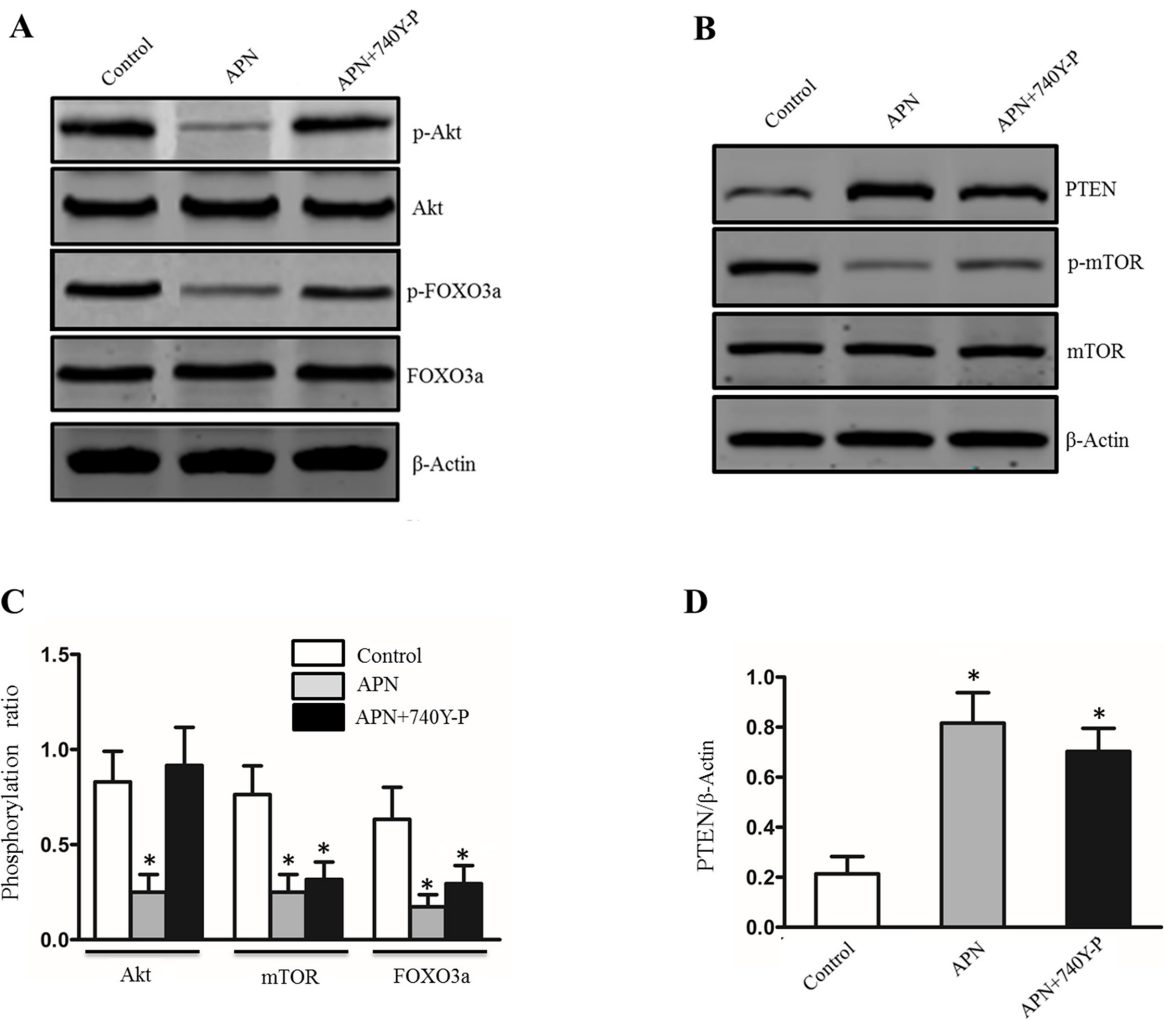
APN induces autophagy in macrophage through Akt-FOXO3a pathway. A, Western blot shows the protein level of p-Akt, Akt, p-FOXO3a, FOXO3a in macrophages stimulated with phosphate buffered saline, APN, Akt agonist (740Y-P) or with APN in combination with 740Y-P. B, Western blot shows the protein level of PTEN, p-mTOR, mTOR in macrophages stimulated with phosphate buffered saline, APN, Akt agonist (740Y-P) or with APN in combination with 740Y-P. C, Quantitative analysis of p-Akt/Akt ratio, p-FOXO3a/FOXO3a ratio and p-mTOR/mTOR ratio in macrophages stimulated with phosphate buffered saline, APN, and APN in combination with 740Y-P, respectively. n = 6 per group. *P<0.05 versus macrophage treated with saline. D, Quantification of the optical density of PTEN in each groups. n = 6 per group. *P<0.05 versus macrophage treated with saline.

To determine how APN modulates autophagy on macrophage, we firstly assessed the activation of Akt/FOXO3a pathway, which has been implicated in autophagy [25]. As shown in Fig 4, APN treatment lessened phosphorylation of the Akt and FOXO3a in macrophage, but there is no difference in the expression of pan Akt and FOXO3a. To further evaluate participation of the Akt/FOXO3a pathway, we employed 740Y-P, a direct activator of Akt pathway [26]. Furthermore, a prior study has demonstrated APN could promote autophagy via modulating PTEN/mTOR pathways [27]. Indeed, APN treatment significantly dampened phosphorylation of mTOR without affecting pan mTOR levels, and enhanced the expression of PTEN in macrophages. However, the change of PTEN/mTOR pathway didn't be abolished after adding 740Y-P. Collectively, these data suggest that the pro-autophagic effects of APN partly depended the Akt/FOXO3a pathway deactivation.

## Discussion

APN has been recognized as an anti-atherosclerotic and anti-inflammatory protein derived from adipocytes. In the present study, we report that PVAT derived-APN suppresses lesions formation after collar-induced carotid atherosclerosis through increasing macrophage autophagy in *ApoE* ^*-/-*^ mice. Furthermore, treatment of macrophages with APN enhanced the expression of the autophagosome marker LC3 through deactivation of Akt/FOX3a signaling pathway.

There is a growing body of evidence highlighting the protective role of APN in cardiovascular diseases [28, 29], especially in atherosclerosis [30]. A study demonstrated that deficiency of APN in *ApoE* ^*−/−*^ mice promotes atherosclerosis and T-lymphocyte accumulation in the atherosclerotic lesions [13]. And, the prior study indicated that adiponectin abates atherosclerosis by reducing oxidative stress or by increasing cholesterol efflux from macrophages [31, 32]. Contrary to data reported here, it has been previously documented that neither genetic overexpression nor APN knockout had any significant effect on atherosclerosis in high-fat fed *ApoE* ^*−/−*^mice [14]. The possible explanation for these differences is the different experimental models. In addition, it is possible that differing derived APN may have contributed to the divergent outcomes. Here, in the perivascular collar placement around carotid artery combined with feeding a high-fat diet *ApoE* ^*−/−*^ mouse model of accelerated atherosclerosis, PVAT-derived APN effectively suppressed collar-induced carotid atherosclerosis. Similar with our results, the other studies have demonstrated that perivascular adipose tissues play a role in the pathogenesis of atherosclerosis in ApoE^-/-^ mice [33, 34]. However, to finally prove our concept, examined the roles of PVAT-derived adiponectin in other model of atherogenesis would be important. This is a limitation of the present study.

Autophagy in atherosclerosis has been extensively investigated with particular focus on vascular smooth muscle cells (SMCs) and endothelial cells (ECs) [35, 36]. The general consensus is that basal autophagy can protect plaque cells against oxidative stress by degrading damaged intracellular material and promoting cell survival [37]. However, accumulating evidences suggest that macrophage autophagy also plays a protective role in advanced atherosclerosis [4, 7]. And, complete deficiency of macrophage autophagy increased vascular inflammation and plaque formation, which was associated with elevated plaque macrophage content [3, 6]. Consistent with these findings, our present study showed that PVAT derived-APN significantly increased autophagy in vascular macrophages in collar-induced carotid atherosclerosis. Moreover, in-vitro experiments indicated that APN induced autophagy in primary macrophage, as evidenced by increased LC3-I processing and Beclin1 expression, which was accompanied by down-regulation of p62. Thus, PVAT derived-APN may act as a key regulator in macrophage activation and the anti-atherosclerotic response.

It has been previously documented that the mTOR signaling pathway negatively regulates autophagy, while Akt activity increases ATP levels and reduces AMPK activity leading to mTOR activation, thereby inhibiting autophagy [38, 39]. A prior study indicated that Akt is an important kinase downstream of the APN pathway and activated by APN via APN receptor [11]. In present study, we observed that treatment of macrophages with APN decreased the phosphorylation of Akt, and more importantly, inhibited the activation of FOX3a gene, which is a key regulator of macrophage autophagy [40]. These results indicate that APN stimulates macrophage autophagy through deactivation of the Akt/FOX3a signaling pathway.

Moreover, data from our current study revealed that APN significantly dampened phosphorylation of mTOR without affecting pan mTOR levels, and enhanced the expression of PTEN in macrophages. These findings are consistent with the crucial role of PTEN/ mTOR in regulation of autophagy. But, activation of Akt pathway by 740Y-P didn't be abolished the change of PTEN/mTOR pathway. Therefore, it appears that PTEN/mTOR signaling pathway could also regulate autophagy at least partially independent from Akt [41]. However, much more experiments would be necessary to identify all the molecular details of the specific pathway.

Besides regulation of autophagy, Akt is also a key cell survival factor with reduced Akt activation directly contributing to apoptosis [42]. Here, our findings reveal that Akt as a main mediator of autophagy that is controlled by APN, but we did not exclude other functions of Akt, which could be carried on in future. It is increasingly clear that the tumor suppressor PTEN is a negative regulator of cell survival [43]. In present study, activation of Akt pathway didn’t affect the expression of PTEN in macrophage after APN treatment. These results showed that APN may promotes apoptosis of macrophage via modulating PTEN/mTOR pathway at least partially independent from Akt. Although autophagy could contribute to cell death under certain experimental settings [44], but the balance between apoptosis and FOXO3a modulated autophagy still needs further study.

This study provides important evidence that PVAT derived-APN exerts profound anti-atherogenic actions to effectively inhibit collar-induced carotid atherosclerosis and increased macrophage autophagy activation in vascular tissue. Furthermore, treatment of macrophages with APN markedly increased Akt/FOX3a activation-mediated autophagy. Our results suggest that APN protects against collar-induced carotid atherosclerosis at least in part through Akt-dependent autophagy activation. The results of the present study may provide a novel therapeutic target against atherosclerosis.
